# 

**Published:** 1886-02

**Authors:** 


### CONTENTS.

### ORIGINAL COMMUNICATIONS—

Some Thoughts and Facts about the Superstitions of Medicine and Mental Influences as Curative Agents. By J. S. Todd, M. D., Atlanta, Ga. . . . . 709

A Case of Compound Comminuted and Depressed Fracture of the Skull—Trephining—Fungus—Recovery. By Howard J. Williams, M. D., Macon, Ga. . . . . 718

A Case of Tuberculosis, Supposed to Have Originated from Arsenious Acid Poisoning. By T. M. Holmes, M. D., Rome, Ga... 723

A Successful Method of Using Jaborandi in Acute and Chronic Bright's Disease. By F. H. O'Brien, M. D., Atlanta, Ga. . . . 729

A Case of Retroflexion of the Uterus Treated by Daily Replacement and the Supporting Tampon. By Virgil O. Hardon, Atlanta, Ga. . . . . 737

Improved Apparatus for Inhalation of Medical Agents. By J. P. Logan, M. D., Atlanta, Ga . . . . . 740

### CLINIC REPORTS—

A Clinical Lecture. By W. S. Elkin, M. D., Atlanta, Ga . . . . . 744

### SOCIETY REPORTS—

Atlanta Society of Medicine . . . . . 749

Chicago Medical Society . . . . . 756

### EDITORIAL—

Death of Professor Joseph A. Eve. . . . . 762
Government Commission for Investigating Yellow Fever Inoculation . . . . . 763
Items . . . . . 728, 761, 767
Necrology . . . . . 768
Roll of Members Medical Association of Georgia, 1885 . . . . . [illegible]
In Memoriam . . . . . [illegible]
Index . . . . . [illegible]

